# Use of angioembolization, treatment modalities and mortality in association with blunt liver trauma in Germany — a data analysis of the TraumaRegister DGU®

**DOI:** 10.1007/s00423-023-03196-6

**Published:** 2023-12-13

**Authors:** Christian Beltzer, Sebastian Imach, Arasch Wafaisade, Rolf Lefering, Benny Kölbel, TraumaRegister DGU

**Affiliations:** 1 Department of General, Abdominal and Thoracic Surgery, German Armed Forces Hospital Ulm, Ulm, Germany; 2 Department of Trauma and Orthopedic Surgery, Cologne-Merheim Medical Center (CMMC), University Witten/Herdecke, Cologne, Germany; 3 Institute for Research in Operative Medicine, Witten/Herdecke University, Cologne, Germany; 4 Committee On Emergency Medicine, Intensive Care and Trauma Management (Sektion NIS) of the German Trauma Society (DGU), Berlin, Germany

**Keywords:** Liver trauma, Angioembolization, Perihepatic packing, Non-operative management, Mortality

## Abstract

**Purpose:**

Angioembolization (ANGIO) is highly valued in national and international guideline recommendations as a treatment adjunct with blunt liver trauma (BLT). The literature on BLT shows that treatment, regardless of the severity of liver injury, can be accomplished with a high success rate using nonoperative management (NOM). An indication for surgical therapy (SURG) is only seen in hemodynamically instable patients. For Germany, it is unclear how frequently NOM $\pm$ ANGIO is actually used, and what mortality is associated with BLT.

**Methods:**

A retrospective systematic data analysis of patients with BLT from the TraumaRegister DGU® was performed. All patients with liver injury AIS ≥ 2 between 2015 and 2020 were included. The focus was to evaluate the use ANGIO as well as treatment selection (NOM vs. SURG) and mortality in relation to liver injury severity. Furthermore, independent risk factors influencing mortality were identified, using multivariate logistic regression.

**Results:**

A total of 2353 patients with BLT were included in the analysis. ANGIO was used in 18 cases (0.8%). NOM was performed in 70.9% of all cases, but mainly in less severe liver trauma (AIS ≤ 2, abbreviated injury scale). Liver injuries AIS ≥ 3 were predominantly treated surgically (64.6%). Overall mortality associated with BLT was 16%. Severity of liver injury ≥ AIS 3, age > 60 years, hemodynamic instability (INSTBL), and mass transfusion (≥ 10 packed red blood cells/pRBC) were identified as independent risk factors contributing to mortality in BLT.

**Conclusion:**

ANGIO is rarely used in BLT, contrary to national and international guideline recommendations. In Germany, liver injuries AIS ≥ 3 are still predominantly treated surgically. BLT is associated with considerable mortality, depending on the presence of specific contributing risk factors.

## Introduction

In blunt abdominal trauma, the liver is one of the most commonly injured organs along with the spleen [1, 2]. Liver trauma is classified into 5 degrees of severity according to AAST (American Association for the Surgery of Trauma) and AIS (Abbreviated Injury Scale; Table 1) [3].

**Table 1:** Liver Injury Scale (2018 revision)

AAST Grade	AIS severity	Imaging criteria (based on CT findings)
I	**2**	Subcapsular hematoma < 10% surface area
		Parenchymal laceration < 1-cm depth
II	**2**	Subcapsular hematoma 10 – 50% surface area; intraparenchymal hematoma < 10 cm in diameter
		Laceration 1–3 cm in depth and ≤ 10-cm length
III	**3**	Subcapsular hematoma > 50% surface area ruptured subcapsular or parenchymal hematoma
		Intraparenchymal laceration > 10 cm
		Laceration > 3 cm depth
		Any injury in the presence of a liver vascular injury or active bleeding contained within liver parenchyma
IV	**4**	Parenchymal disruption involving 25 – 75% of a hepatic lobe
		Active bleeding extending beyond the liver parenchyma into the peritoneum
V	**5**	Parenchymal disruption > 75% of hepatic lobe
		Juxtahepatic venous injury to include retrohepatic vena cava and central major hepatic veins

*AAST* American Association for the Surgery of Trauma, *AIS* Abbreviated Injury Score. Active bleeding from a vascular injury presents as vascular contrast, focal, or diffuse that increases in size or attenuation in delayed phase; more than one grade of liver injury may be present and should be classified by the higher grade of injury [3]

The possible treatment options for blunt liver trauma (BLT) range from nonoperative management (NOM), which can be supported by interventional angioembolization (ANGIO; transcutaneous angiography and embolization, e.g., coiling, of arterial liver bleeding through a catheter that is inserted in the femoral or brachial artery), to a damage-control surgery (DCS; e.g., midline laparotomy with perihepatic packing and temporary abdominal closure) approach [4, 5]. It has been demonstrated that NOM of hemodynamically stable patients with BLT can be performed safely in most cases [6], and as a consequence a paradigm shift in the treatment has evolved from mandatory surgical therapy (SURG) to NOM [6–9]. In principle, NOM can be performed also for higher-grade liver injuries with a high success rate of > 95% [10, 11]. Regardless of the grade of liver injury, the hemodynamic status of the patient is considered the most important decision criterion for selecting treatment in terms of NOM vs. SURG [12–15].

The high value of ANGIO in BLT is emphasized in national (German S3 Guideline Polytrauma) [16] and international guidelines (Liver Trauma: World Society of Emergency Surgery/WSES-Guideline) [17].

However, for the treatment of BLT in Germany, it is unclear how often ANGIO is actually used, which treatment modalities (NOM vs. SURG) are performed, and with which mortality BLT is associated.

## Material and methods

### Aims and objectives of the study

The primary purpose of the study was to evaluate the frequency of ANGIO use in conjunction with BLT.

Other objectives were the analysis of:

- Treatment modalities (NOM, SURG) and frequency of use according to AIS
- In-hospital-mortality of BLT depending on the severity of liver injury (AIS) as well as on the treatment performed (NOM versus SURG)
- Correlation of hemodynamic instability (INSTBL, defined as any transfusion requirement and/or systolic blood pressure ≤ 90 mmHg on arrival) with AIS severity of liver injury
- Association of BLT with abdominal and extra-abdominal concomitant injuries
- Surgical procedures (suture hepatorrhaphy/HR, perihepatic packing/PHP, partial resection/RES) used among patients with SURG
- Ranking of risk factors according to their influence on mortality in BLT, including the treatment strategy

### Analysis

A retrospective systematic analysis of patients with BLT in the database of the TraumaRegister DGU® was performed (standard data set).

**Fig. 1:**
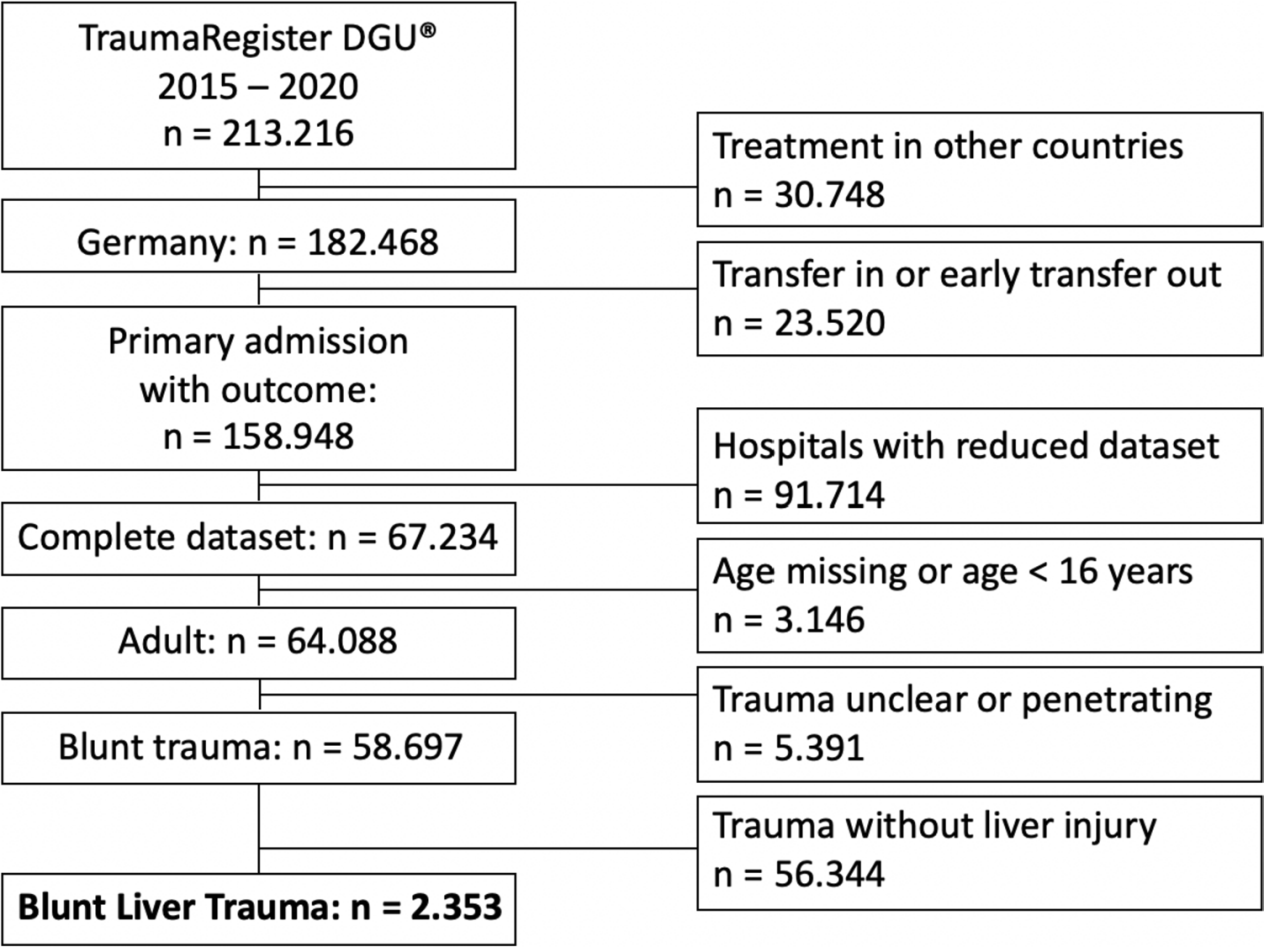
Flowchart of systematic review of the “TraumaRegister DGU® database” with criteria for patient inclusion and exclusion

Only patients treated in German hospitals were considered. Patients with penetrating injuries, patients < 16 years of age, patients treated in hospitals which use the reduced data set (limited information about the surgical approach), and transferred patients were excluded. The time period was limited from 2015 to 2020 (for details see Fig. 1).

Demographic data (age, sex) and relevant concomitant injuries (head injury AIS ≥ 3, thoracic injury AIS ≥ 3, abdominal injuries AIS ≥ 3 and extremity injury AIS ≥ 3) were collected for all patients to control for potential confounders with influence on potential differences between treatment groups.

For all patients with BLT, we assessed in how many cases ANGIO was performed, and whether therapy was by NOM or SURG. For analysis of surgical procedures only search terms and procedure codes related to liver trauma were used.

Coagulopathy was defined as Quick < 60% or partial thromboplastin time (PTT) ≥ 40 s or international normalized ratio (INR) ≥ 1.4.

It was evaluated for patients with NOM and SURG in how many cases packed red blood cells/pRBC (< 10 pRBC or mass transfusion ≥ 10 pRBC) were given, and in how many cases INSTBL was prevalent.

### Statistical tests and methods

Differences between patients with NOM and SURG were tested using chi-squared test, or Fischer’s exact test where possible. For ordinal and continuous data Mann–Whitney-*U*-Test was used.

To evaluate the influence of liver injury severity (AIS) on overall mortality, on hemodynamic instability (INSTBL) and on the frequency of surgical treatment (SURG), the chi-squared test for trend was used. For all tests performed, a *p*-value < 0.05 was considered significant.

The parameters age, severity of liver trauma (AIS 2–5), INSTBL, surgical management (SURG) and mass transfusion ≥ 10 pRBC, were assessed for their adjusted risk of mortality in patients with liver trauma using multivariate logistic regression analysis. Adjusted effects were presented as odds ratios (OR) with 95% confidence intervals.

All calculations were performed using SPSS Statistics Version 25 (IBM, Armonk, NY, USA). Diagrams were created using GraphPad Prism Version 9 (GraphPad Software, San Diego, CA, USA).

### TraumaRegister DGU®

The TraumaRegister DGU® of the German Trauma Society (Deutsche Gesellschaft für Unfallchirurgie, DGU) was founded in 1993. The aim of this multi-center database is a pseudonymized and standardized documentation of severely injured patients.

Data are collected prospectively in four consecutive time phases from the site of the accident until dis-charge from hospital: (A) pre-hospital phase, (b) emergency room and initial surgery, (c) intensive care unit, and (d) discharge. The documentation includes detailed information on demographics, injury pattern, comorbidities, pre- and in-hospital management, course on intensive care unit, relevant laboratory findings including data on transfusion, and outcome of each individual. The inclusion criterion is admission to hospital via emergency room with subsequent ICU/ICM care or reach the hospital with vital signs and die before admission to ICU. The infrastructure for documentation, data management, and data analysis is provided by AUC – Academy for Trauma Surgery, a company affiliated to the German Trauma Society (DGU). The scientific leadership is provided by the Committee on Emergency Medicine, Intensive Care and Trauma Management (Sektion NIS) of the German Trauma Society. The participating hospitals submit their data pseudonymized into a central database via a web-based application. Scientific data analysis is approved according to a peer review procedure laid down in the publication guideline of TraumaRegister DGU®. The participating hospitals are primarily located in Germany (90%), but a rising number of hospitals of other countries contribute data as well (at the moment from Austria, Belgium, China, Finland, Luxembourg, Slovenia, Switzerland, The Netherlands, and the United Arab Emirates). Currently, about 28.000 cases from almost 700 hospitals are entered into the database per year. Participation in TraumaRegister DGU® is voluntary. For hospitals associated with TraumaNetzwerk DGU®, however, the entry of at least a basic data set is obligatory for reasons of quality assurance.

The study was performed following the current publication guideline of the TraumaRegister DGU® and is registered as TR-DGU Project ID 2022–013.

## Results

### Demographic and clinical data

A total of 2353 patients with Blunt Liver Trauma (BLT) met the inclusion criteria and were enrolled in the analysis. AIS 2 severity was present in 1763 patients (74.9%), which was the most common among all AIS severity levels. The prevalence was less with increasing AIS-values, and only 2.7% had AIS 5/6.

Compared with NOM, the SURG group contained significantly more patients with AIS severity levels 3–6 (severe liver trauma; *p*-values provided in Table 2). The mean ISS was 29.7 ± 16.0.

**Table 2:** Demographic and clinical data of patients with blunt liver trauma

Parameter	Overall (*n* = 2353)	NOM (*n* = 1669)	SURG (*n* = 684)	*p*-value
Age (mean ± SD)	42.8 ± 18.9	43.2 ± 19.0	41.7 ± 18.5	0.092
Male, *n* [%]	1.497 [63.6]	1062 [63.6]	435 [63.6]	1.00
ISS (mean ± SD)	29.7 ± 16.0	27.1 ± 15.2	36.0 ± 16.2	< 0.001
Severity of liver injury				< 0.001
AIS 2, *n* [%]	1.763 [74.9]	1460 [87.5]	303 [44.3]	
AIS 3, *n* [%]	360 [15.3]	149 [8.9]	211 [30.8]	
AIS 4, *n* [%]	167 [7.1]	42 [2.5]	125 [18.3]	
AIS 5/6, *n* [%]	63 [2.7]	18 [1.1]	45 [6.6]	
Concomitant injuries (AIS ≥ 3)*:				
Head, *n* [%]	739 [31.4]	511 [30.6]	228 [33.3]	0.204
Thorax, *n* [%]	1662 [70.6]	1.137[68.1]	525 [76.8]	< 0.001
Extremities, *n* [%]	965 [41.0]	637 [38.2]	328 [48.0]	< 0.001
Spleen, *n* [%]	319 [13.6]	135 [8.1]	184 [26.9]	< 0.001
Stomach/bowel, *n* [%]	110 [4.7]	44 [2.6]	66 [9.6]	< 0.001
Pancreas, *n* [%]	37 [1.6]	13 [0.8]	24 [3.5]	< 0.001
Kidney, *n* [%]	124 [5.3]	65 [3.9]	59 [8.6]	< 0.001
Vessels, *n* [%]	153 [6.5]	88 [5.3]	65 [9.5]	< 0.001
Blood transfusion				< .001
1–9 pRBC, *n* [%]	725 [31.0]	299 [18.0]	246 [36.2]	
Mass Transfusion ≥ 10 pRBC, *n* [%]	180 [7.6]	66 [4.0]	114 [16.8]	
Systolic BP ≤ 90 mmHg, *n* [%]	460 [20.6]	257 [16.2]	203 [31.7]	< 0.001
Hemodynamic Instability*, *n* [%]	894 [38.0]	478 [28.6]	416 [60.8]	< 0.001
Coagulopathy (Quick < 60% or PTT ≥ 40 s. or INR ≥ 1.4)	585 [26.2]	327 [20.7]	258 [39.8]	< 0.001
CT Imaging, *n* [%]	2254 [95.8]	1613 [96.6]	641 [93.7]	0.002

*AIS* abbreviated injury score, *BP* blood pressure, *CT* computed tomography, *INR* international normalized ratio, *INSTBL* hemodynamic instability (defined as systolic blood pressure ≤ 90 mmHg on arrival and/or any transfusion requirement), *ISS* Injury Severity Score, *pRBC* packed red blood cells, *PTT* partial thromboplastin time, *SD* standard deviation, * there may be several concomitant injuries per patient

The average age of the patients was 42.8 years, and 1497 of all patients (63.6%) were male. For both parameters, there were no statistically significant differences between patients with NOM or SURG.

A total of 725 patients (31.0%) required transfusions (packed red blood cells, pRBC), and among them 180 patients (7.6%) received a mass transfusion (≥ 10 pRBC).

INSTBL was present in 894 patients (38.0%), with a significantly higher incidence in the SURG group compared with NOM (SURG: 60.8% vs. NOM: 28.6%, *p* < 0.001).

Coagulopathy was seen in 26.2% of cases and was significantly more common in patients receiving surgical therapy (SURG 39.8% vs. NOM 20.7%, *p* < 0.001).

Concomitant thoracic injuries ≥ AIS 3 were present in 1662 cases (70.6%; NOM: 68.1% vs. SURG: 76.8%,* p* < 0.001) and were the most frequent, followed by extremity injuries AIS ≥ 3 (*n* = 965, 41.0%; NOM: 38.2% vs. SURG: 48.0%, *p* < 0.001) and head injuries AIS ≥ 3 (*n* = 739, 31.4%; NOM: 30.6% vs. SURG: 33.3%, *p* = 0.204).

Splenic injuries ≥ AIS 3 were the most frequent concomitant abdominal injuries (*n* = 319, 13.6%; NOM: 8.1% vs. SURG: 26.9%), and stomach/bowel-injuries ≥ AIS 3 were present in 4.7% (NOM: 2.6% vs. SURG: 9.6%).

Computed tomography (CT) was performed in 2254 patients (95.8%). Details of demographic data and clinical parameters are shown in Table 2.

### ANGIO use and treatment modalities according to AIS severity of liver injury

**Fig. 2:**
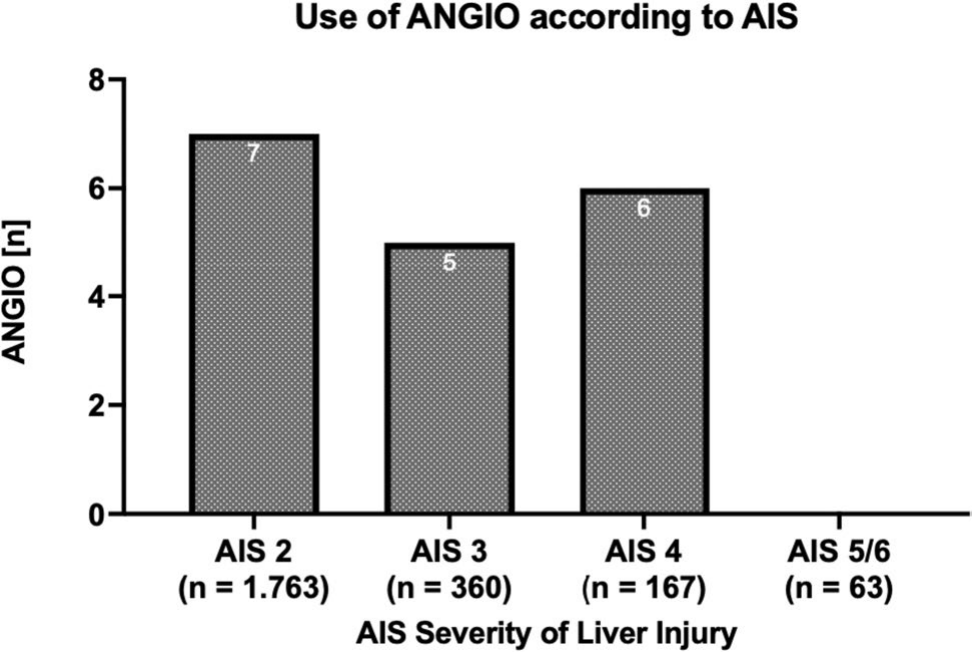
Use of ANGIO according to AIS Severity of Liver Injury; total use of ANGIO (AIS 2–6): *n* = 18; AIS = abbreviated injury score, ANGIO = angiography ± embolization

ANGIO was used in 18 of 2353 cases (0.8%), most often at severity AIS 4 (*n* = 6 of 167, 3.6%; Fig. 2).

**Fig. 3:**
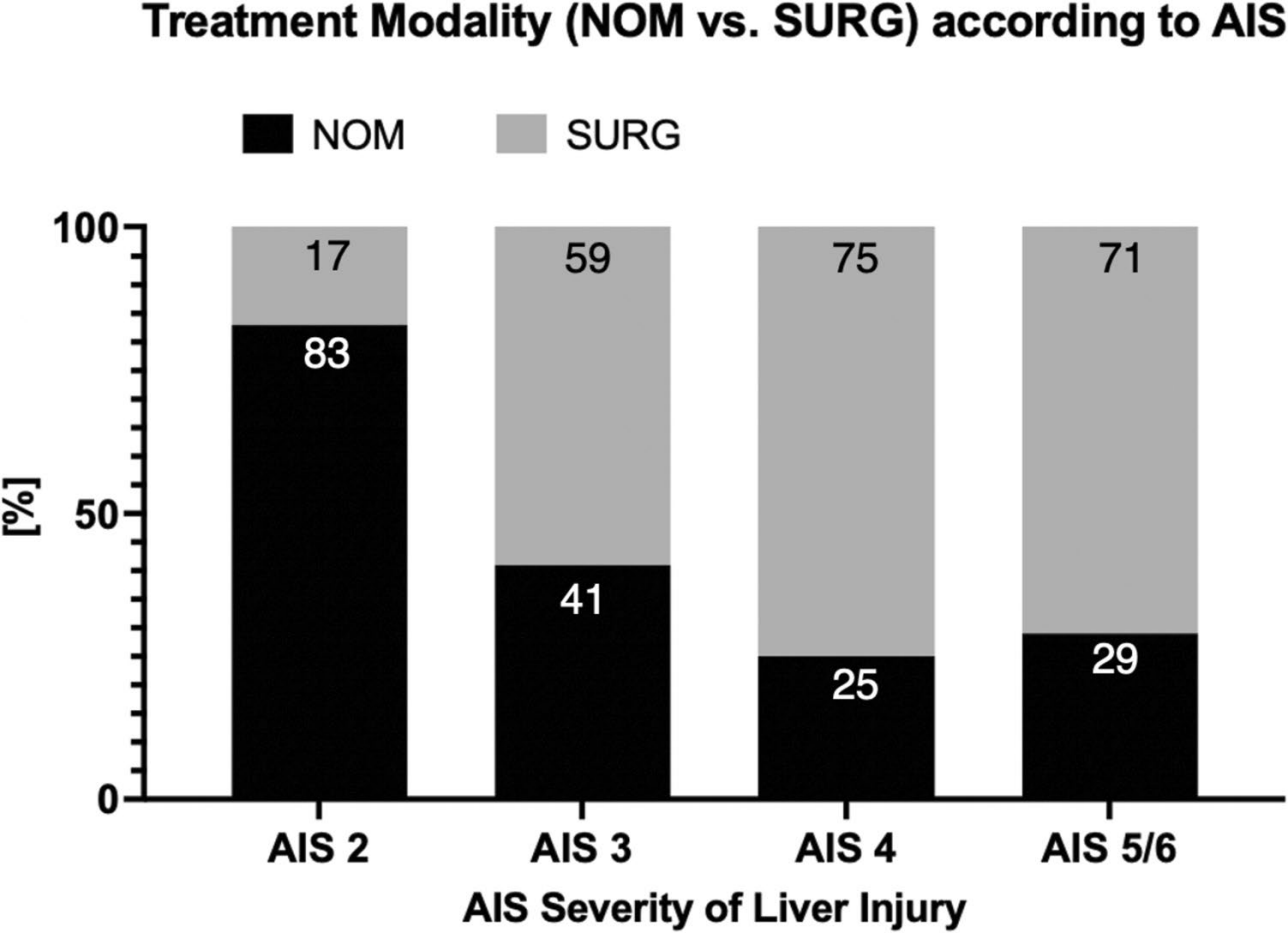
Treatment modality (NOM vs. SURG) according to AIS Severity of Liver Injury, AIS = abbreviated injury score, NOM = non-operative management, SURG = surgery, *chi-square-test for trend (rising of treatment modality SURG with increasing AIS): *p* < 0.0001

Overall, treatment of patients with BLT was mainly performed by NOM (*n* = 1669 of 2353, 70.9% vs. SURG: *n* = 684 of 2353, 29.1%). For severity grades AIS 3–6, therapy was predominantly performed using SURG (AIS 3: *n* = 211 of 360, 58.6%; AIS 4: *n* = 125 of 167, 74.9%; AIS 5/6: *n* = 45 of 63, 71.4%; AIS 3–6: 381 of 590, 64.6%). With increasing AIS injury severity, therapy using SURG also increased significantly (chi-square-test for trend, *p* < 0.001; Fig. 3).

### Surgical procedures according to AIS severity of liver injury

**Fig. 4:**
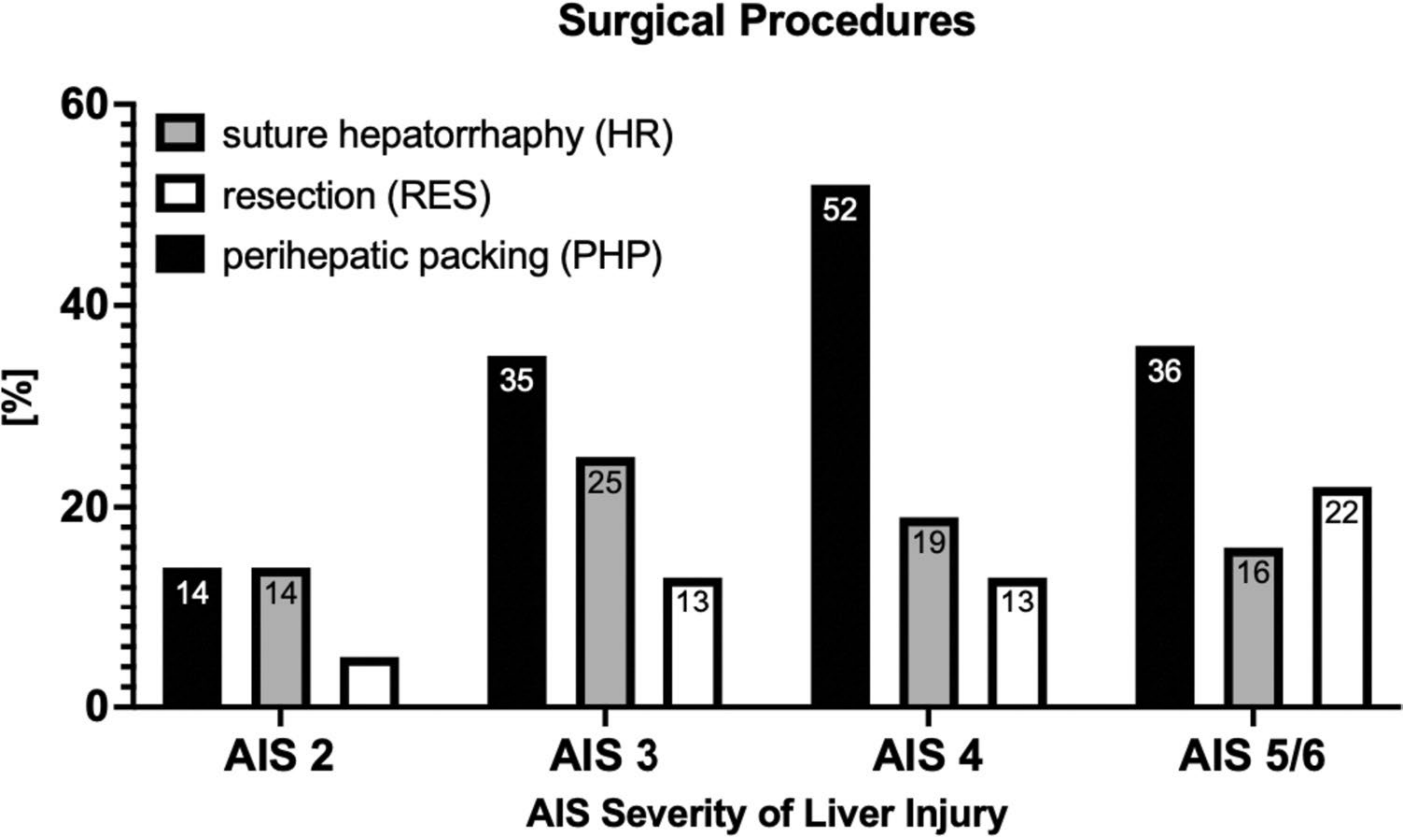
Surgical procedures in patient cohort with surgery for liver trauma according to AIS severity of liver injury; AIS = abbreviated injury score; more than one surgical procedure may have been applied per patient

Among specific surgical procedures in the SURG group, perihepatic packing (PHP) was used most frequently overall (AIS 2–6: *n* = 197 of 684, 29%). Of these, PHP was applied most often at AIS 4 (*n* = 65 of 125, 52%), and least frequently at AIS 2 (*n* = 43 of 303, 14%). Suture hepatorrhaphy (HR) was performed second most frequently overall (AIS 2–6: *n* = 126 of 684, 18%). Of the surgical procedures, liver resections (= RES) were used least commonly (*n* = 67 of 684, 10%), with the highest use at AIS 5/6 (*n* = 10 of 45, 22%) (Fig. 4).

### Mortality and hemodynamic instability according to AIS severity of liver injury

Overall mortality was 16% (AIS 2–6: *n* = 377 of 2353). There was a statistically significant increase in mortality depending on the AIS (chi-square-test for trend: *p* < 0.0001). Mortality was lowest in AIS 2 (*n* = 198 of 1763, 11%) and highest in AIS 5/6 (*n* = 39 of 61, 62%). For AIS 3–6 (severe liver trauma), mortality was 33% (*n* = 179 of 590).

**Fig. 5:**
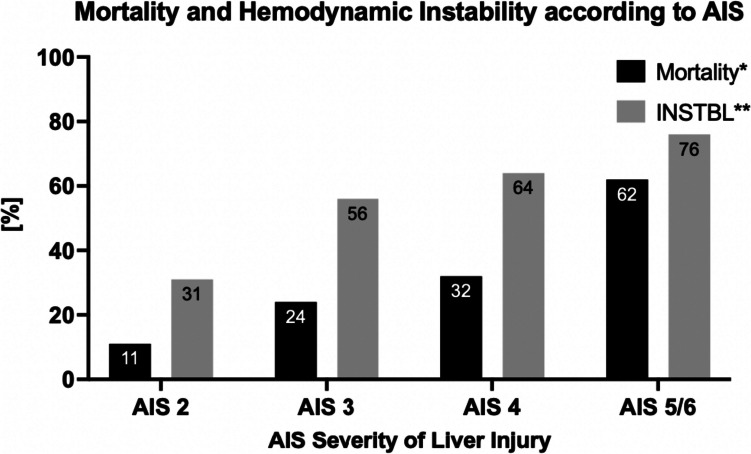
Mortality and hemodynamic instability according to AIS severity of liver injury, AIS = abbreviated injury score, INSTBL = hemodynamic instability (defined as systolic blood pressure ≤ 90 mmHg on arrival and/or any transfusion requirement), *chi-square-test for trend (rising mortality with increasing AIS): p < 0.0001, **chi-square-test for trend (rising INSTBL with increasing AIS): *p* < 0.0001

Among 2353 patients with BLT, 894 (38%) showed hemodynamic instability (INSTBL). A statistically significant increase with rising AIS was also present for INSTBL (chi-square-test for trend, *p* < 0.0001), with the highest incidence at AIS 5/6 (*n* = 48 of 63, 76%) (Fig. 5).

### Mortality, hemodynamic instability, coagulopathy, and mass transfusion according to treatment (NOM vs. SURG)

**Fig. 6:**
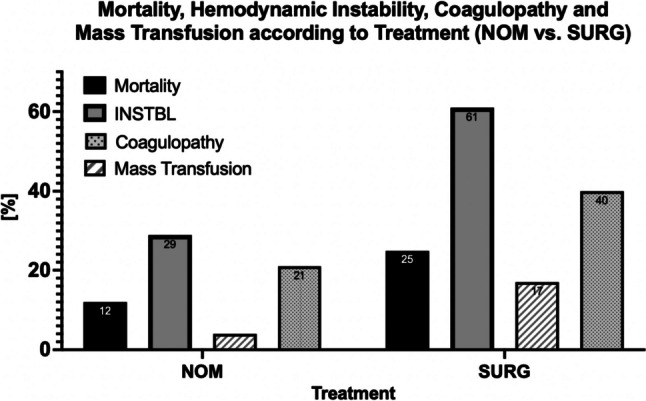
Mortality, hemodynamic instability, coagulopathy, and mass transfusion (defined as transfusion ≥ 10 pRBC) according to treatment (NOM vs. SURG); AIS = abbreviated injury score, coagulopathy = Quick < 60% or partial thromboplastin time (PTT) ≥ 40 s or international normalized ratio (INR) ≥ 1.4, INSTBL = hemodynamic instability (defined as systolic blood pressure ≤ 90 mmHg on arrival and/or any transfusion requirement), NOM = non-operative management, SURG = surgery; all differences NOM vs. SURG were statistically significant (*p* < 0.001, chi-squared test)

Mortality was 12.3% (*n* = 206 of 1669) in the group with NOM and 25.0% (*n* = 171 of 684) in SURG. This difference was statistically significant (chi-square test, *p* < 0.001). In the group with treatment NOM, 478 of 1669 patients (29%) were hemodynamically instable (INSTBL), compared with 416 of 684 patients (61%) with SURG, and this difference was also statistically significant (chi-square test, *p* < 0.001). Patients in the SURG group were significantly more likely to have coagulopathy (SURG: 39.8% vs. NOM: 20.7%, *p* < 0.001). Patients with SURG required mass transfusions (≥ 10 pRBC) in 16.8% vs. 4.0% in patients with NOM (*p* < 0.001) (Fig. 6).

### Independent risk factors contributing to mortality

**Fig. 7:**
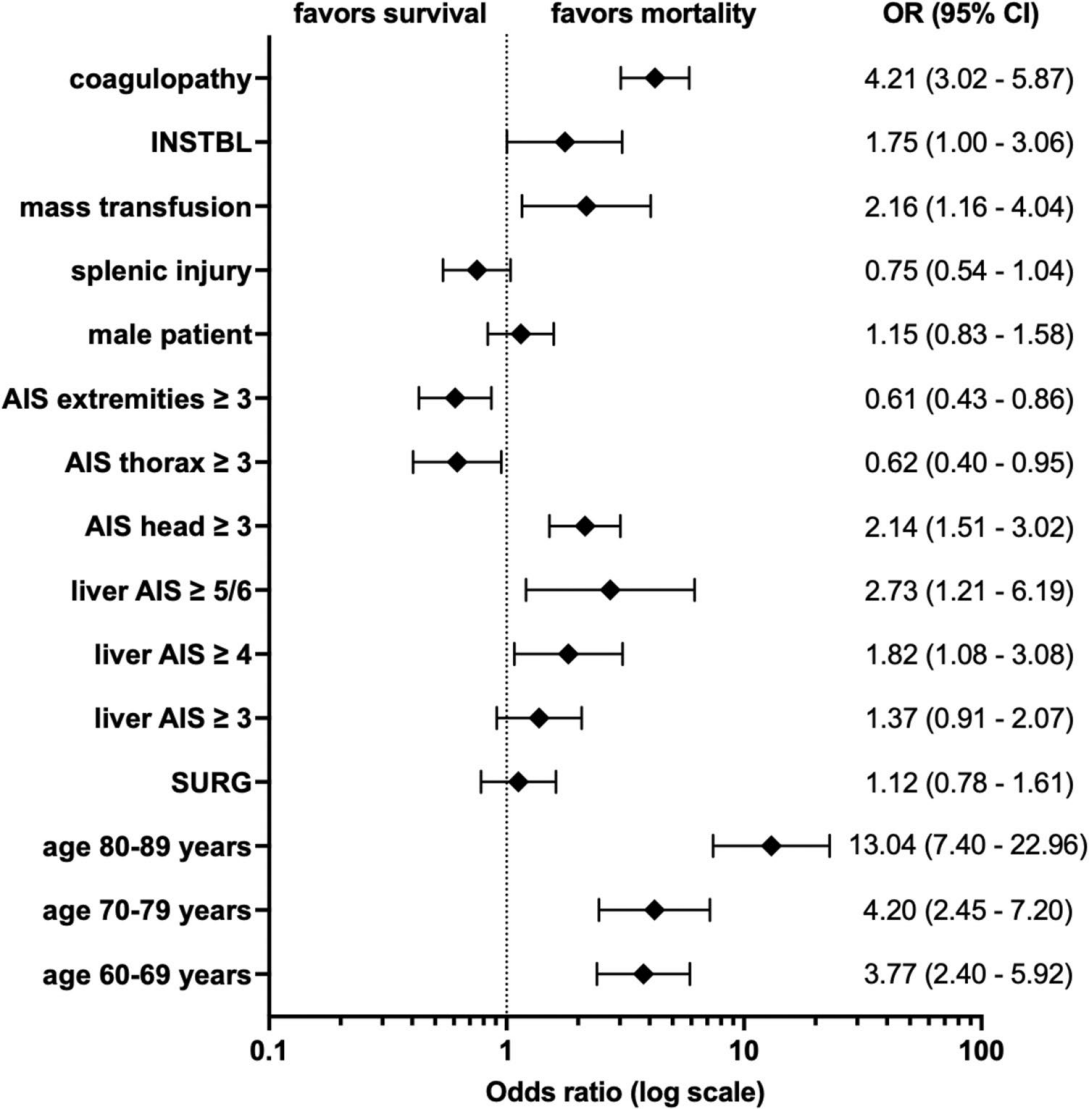
Logistic regression analysis in patients with blunt liver trauma, with hospital mortality as dependent variable. Adjusted odds ratios (OR) are presented with 95% confidence intervals (CI). AIS = abbreviated injury score, coagulopathy = Quick < 60% or partial thromboplastin time (PTT) ≥ 40 s or international normalized ratio (INR) ≥ 1.4, INSTBL = hemodynamic instability (defined as systolic blood pressure ≤ 90 mmHg on arrival and/or any transfusion requirement), SURG = surgery

The strongest risk factor for mortality was age 80—89 years (OR: 13.04, 95% CI: 7.40 –22.96), followed by coagulopathy (OR: 4.21, 95% CI: 3.02 – 5.87), age 70 – 79 years (OR: 4.20, 95% CI: 2.45 – 7.20), age 60 – 69 years (OR: 3.77, 95% CI: 2.40 – 5.92), liver AIS ≥ 5/6 (OR: 2.73, 95% CI: 1.21 – 6.19) and mass transfusion ≥ 10 pRBC (OR: 2.16, 95% CI: 1.16 – 4.04). INSTBL was also associated with increased mortality (OR: 1.75, 95% CI: 1.00 – 3.06). Surgical treatment (SURG) showed an OR of 1.12, and concomitant splenic injury did not contribute to increased mortality (OR: 0.75; for other risk factors contributing to mortality, odds ratios and 95% confidence intervals, see Fig. 7).

## Discussion

The aim of our study was to analyze the frequency of use of ANGIO in BLT in Germany, as suggested by current guidelines [16, 17].

Therapeutic modalities (NOM vs. SURG; frequency of specific surgical procedures used) and mortality were also to be evaluated.

Furthermore, independent risk factors should be ranked for their adjusted risk of mortality and therapy selection.

Of note, in comparison to the estimated high value of ANGIO in BLT in the international literature [18–20], it is hardly established in Germany so far, which is underlined by a rare application of only 0.8% in our study.

Data on the frequency of use of ANGIO vary in the literature. Gaarder et al. reported the usage of ANGIO in 44% of patients with liver trauma after establishing a treatment protocol for NOM [21]. Tignanelli et al. found differences of ANGIO usage dependent on the level of trauma center (12% in level 1 versus 5% in Level 2) [22].

Comparison of our study results with other multicenter evaluations of large patient cohorts appears interesting here. In a “National Trauma Data Bank analysis” (USA) including 6409 patients with grade 4 and 5 liver injuries, ANGIO was used in 11% [23].

It should not be left unmentioned that low use of ANGIO < 1% in BLT has also been reported by other authors [7].

However, reasons for the low use of ANGIO in BLT in Germany in comparison to the above given percentage of usage in the USA remain unclear and cannot be answered conclusively. Firstly, it should be emphasized that liver trauma usually results in venous or portal venous bleeding, which cannot be addressed by arterial angioembolization.

Secondly, specific structural and personnel requirements are necessary to offer diagnostics and therapy by means of ANGIO on a 24-h basis, which is not the case with all trauma centers.

Thirdly, ANGIO is often used as an adjunct therapy to NOM, although benefit is not clearly proven, especially in stable patients with liver trauma [24].

The lack of standardized treatment protocols for the management of BLT in many hospitals and possible complications associated with ANGIO (e.g., liver necrosis, infarction, abscess formation) [19, 25, 26] may be further explanations for the low frequency of its use. Therapeutic failures with ANGIO require immediate surgical exploration, which also depends on the existing hospital structure in terms of personnel and equipment.

In our own cohort, patients with BLT were predominantly (71%) treated by NOM. Subgroup analysis according to AIS showed that NOM was used mainly with liver injuries AIS ≤ 2. In AIS 3–6 (severe liver trauma), surgical therapy (SURG) dominated in our patients.

In a study by Barbier et al. including 116 patients with BLT and a mean AIS of 2.9, NOM was used in 80 patients (69%) with a success rate of 96% [27].

It is well known that the generally high success rate of NOM decreases with increasing AIS [28]. A study published by Gourgiotis et al. showed, while treatment with NOM was used in 71% for AAST ≤ 2, this was only the case for 5% with AAST ≥ 3 [29].

In contrast, Christmas et al. reported the predominant use of NOM for grades AAST 1–4 (NOM in AAST 1 = 82%; 2 = 63%; 3 = 63%; 4 = 61%; 5 = 32%) [30]. Gaski et al. demonstrated that after implementation of a management algorithm and a massive hemorrhage protocol for liver trauma, consisting of tranexamic acid and plasma, platelets, and pRBC in a 1:1:1 ratio with defined goals of Hb (8–10 g/dl), INR (< 1.5) and platelets (> 100 × 10^9^/l), NOM was also used in AAST 4/5 at 70%, with a 98% success rate [31].

Due to the retrospective design of our study, it is not possible to distinguish between “responders” and “non-responders” to resuscitation, which is an important criterion for therapy selection (NOM vs. SURG) [15].

Another major factor influencing treatment selection (NOM vs. SURG) and outcome may be concomitant abdominal injuries AIS ≥ 3, which by themselves may be an indication for surgery (e.g. splenic or bowel injuries). The SURG group showed significantly higher rates of relevant concomitant abdominal injuries.

PHP was the most commonly used of all surgical procedures (> HR > RES) in our patients. PHP is the treatment of choice in the hemodynamically unstable patient with liver injury for hemorrhage control [32, 33]. Keizer et al. analyzed the most commonly used procedures in patients with SURG for BLT [34]. Among these, PHP and HR were most frequently used (PHP: 31.5% > HR 12.6% > RES: 1.4%). Liver resections are rarely required in the initial surgical treatment of liver trauma. Anatomical liver resections, which may be necessary in individual cases, should be performed by experienced liver surgeons to achieve acceptable outcome [35–37].

Interestingly, existing coagulopathy was found to be the second strongest independent factor influencing mortality in BLT. Therefore, a second hit by SURG should be avoided, particularly in patients with coagulopathy (and without INSTBL), as any unnecessary surgical treatment might result in worse outcome. Rotondo et al. already described coagulopathy as a component of the lethal triad in 1997, along with acidosis and hypothermia [38]. These are considered triggers to an approach according to the principles of damage control surgery (DCS). To our knowledge, our study was the first to analyze the association between coagulopathy and mortality in BLT.

The parameters INSTBL, mass transfusion and coagulopathy influence or reinforce each other, so that an isolated consideration can only be made cautiously.

In-hospital-mortality associated with blunt liver trauma in our study was 16% and increased significantly with the severity of liver injury (11% in AIS 2 and up to 62% in AIS 5/6). The influence of liver injury severity and INSTBL on mortality has been described previously [39].

Patients with SURG were at higher risk of mortality in our analysis. This was found to be due to higher rates of coagulopathy, liver injuries AIS ≥ 3, INSTBL and mass transfusions ≥ 10 pRBC in patients with SURG. SURG could not be identified as a relevant independent risk factor contributing to mortality.

Reported mortality of BLT in the literature is subject to considerable variation. Other studies on BLT have found a mortality of 7.8% [11], 9.1% [34], 13% [39], 15.4% [22], and 25.4% [40]. In this context, the heterogeneity of the investigated patient cohorts between the studies (e.g., severity liver injury, concomitant injuries, ISS) has to be taken into account, which makes a comparability of data and results difficult. The high overall injury severity of the patients in our analysis is also reflected by the high ISS of 29.7. Regarding the independent factors influencing mortality, there are apparently protective effects for extremity and thoracic injuries. It should be noted that these injuries occur very frequently in absolute numbers, and are thus also associated proportionally more with low-grade liver injuries and NOM — and thus lower mortality.

Irrespective of this, we were able to show that BLT in Germany is associated with considerable mortality, and what the main independent risk factors are in this regard.

The strength of the study is its multicenter evaluation and the high number of patients included. In contrast to many single-center studies, statements can thus be made about the reality of BLT treatment and outcomes in Germany. The retrospective study design must be considered a major limitation.

## Conclusion

In Germany, ANGIO is rarely used in severely injured patients with BLT compared with the published results of other authors (use of only 0.8%). Surgical treatment is predominant in liver injuries with an AIS ≥ 3, because injuries of this severity are often associated with abdominal and extra-abdominal concomitant injuries and patients are commonly hemodynamically unstable. BLT is associated with a significant mortality of 16%. In-hospital mortality and choice of treatment (NOM vs. SURG) are highly dependent on the severity of liver injury (AIS), any coagulopathy present, and the hemodynamic status of the patient.

## Data Availability

Datasheets and analysis are made available upon request to the corresponding author.
